# Copy Number Variation and Osteoporosis

**DOI:** 10.1007/s11914-023-00773-y

**Published:** 2023-02-16

**Authors:** Nika Lovšin

**Affiliations:** University of Ljubljana, Faculty of Pharmacy, Aškerčeva 7, 1000 Ljubljana, Slovenia

**Keywords:** Copy number variation, Osteoporosis, Bone mineral density, Fragility fractures, Structural variations

## Abstract

**Purpose of Review:**

The purpose of this review is to summarize recent findings on copy number variations and susceptibility to osteoporosis.

**Recent Findings:**

Osteoporosis is highly influenced by genetic factors, including copy number variations (CNVs). The development and accessibility of whole genome sequencing methods has accelerated the study of CNVs and osteoporosis. Recent findings include mutations in novel genes and validation of previously known pathogenic CNVs in monogenic skeletal diseases. Identification of CNVs in genes previously associated with osteoporosis (e.g. *RUNX2*, *COL1A2*, and *PLS3*) has confirmed their importance in bone remodelling. This process has been associated also with the *ETV1-DGKB*, *AGBL2*, *ATM*, and *GPR68* genes, identified by comparative genomic hybridisation microarray studies. Importantly, studies in patients with bone pathologies have associated bone disease with the long non-coding RNA *LINC01260* and enhancer sequences residing in the *HDAC9* gene.

**Summary:**

Further functional investigation of genetic loci harbouring CNVs associated with skeletal phenotypes will reveal their role as molecular drivers of osteoporosis.

## Osteoporosis and Genetic Variations

Osteoporosis (OP) is a complex disease that is highly influenced by genetic factors. It is estimated that it is 50 to 80% hereditary [1–3]. Genetic susceptibility to osteoporosis has been extensively studied [1, 2, 4]. One of the most important risk factors for the development of OP is a positive family history underlying the importance of genetic factors in the development of disease [1, 5–8]. In addition to genetic factors, old age, female sex, and low mineral density are the strongest determinants of osteoporosis [9, 10]. Age-related bone loss increases the risk for bone fractures due to bone fragility and propensity for falls [11]. Osteoporotic fractures are the biggest impact that osteoporosis has on socioeconomics. It is estimated that osteoporosis affects 200 million people worldwide, and in the USA alone, the cost of treating osteoporotic fractures is estimated at $17 billion (reviewed in [2, 12]). Genetic variants that contribute to development of complex diseases are characterized by their low penetrance and high allele frequency. Based on nucleotide composition, genetic variations are divided into two groups: Single nucleotide polymorphisms (SNPs) and structural variations. SNP variations in OP have been studied in detail in several world populations over the past 15 years [5, 12, 13]. Analyses of SNP variations are usually performed using microarrays as part of genome-wide association studies (GWAS). In fact, most disease association studies have focused on the analysis of SNPs in GWAS [5, 7, 12, 13]. Structural variations in otherwise healthy people have also been recognized as possible causes of heritable complex diseases, such as OP [14]. Structural variations are any type of variations that alter chromosome structure and are defined as changes larger than 50 bp [15, 16]. Structural variations comprise inversions, insertions, translocations and genomic unbalances (deletions and duplications) that contribute to changes in the DNA amount [17]. Telomeric regions showed a higher rate of structural variation [18]. Large chromosomal changes have been recognized as disease causing for a long time (e.g. Down syndrome) [19], yet it was only after development of new sequencing technologies that smaller chromosomal modifications were also recognized as disease causing structural variations [20].

## Copy Number Variations and Osteoporosis

**Fig. 1:**
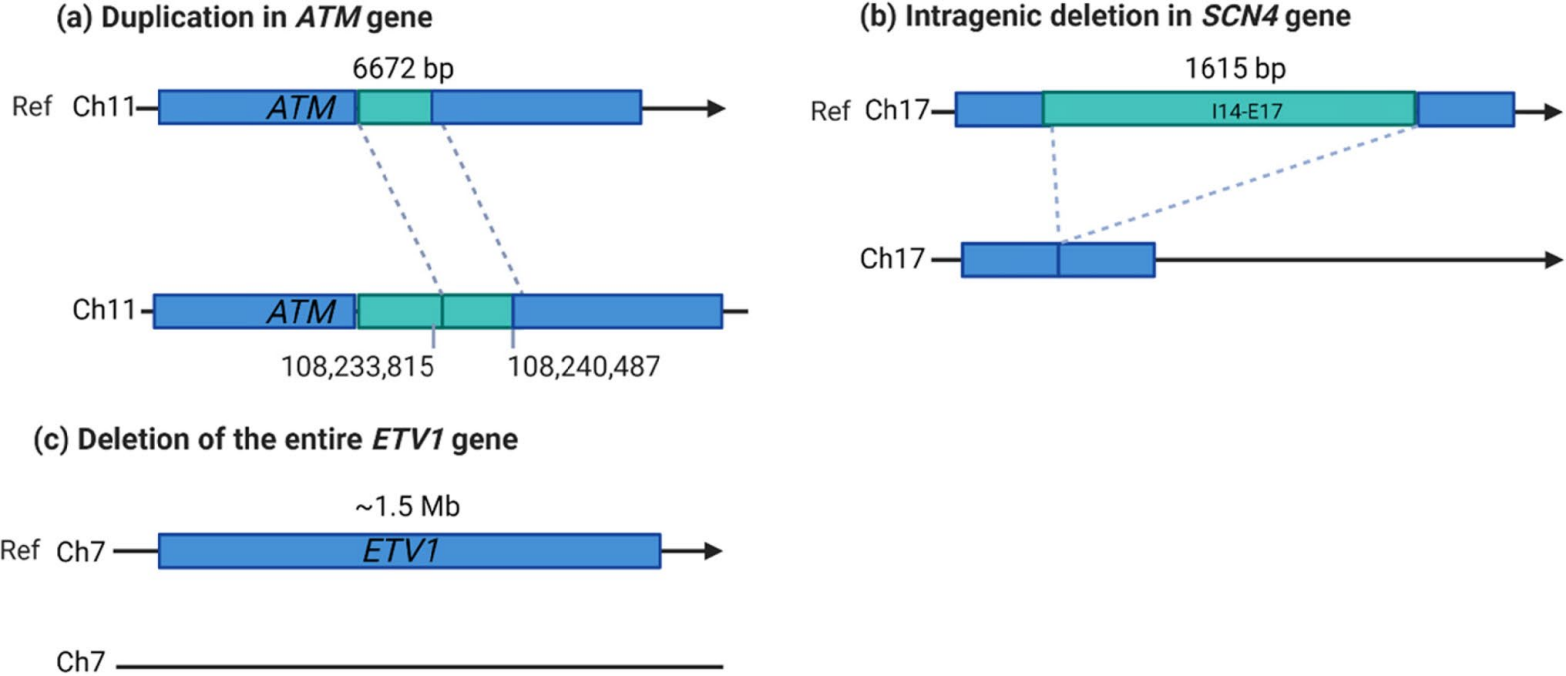
Schematic representation of copy number variation in **a** *ATM* gene (duplication), **b** *SCN4A* gene (deletion of intragenic region) and **c** *ETV1* gene (deletion of a gene). Adapted from [34•]. Created with BioRender.com

Copy number variations (CNVs) are a subtype of structural variations characterized by deletions and duplications of DNA segments larger than 50 bp [21, 22]. CNVs contribute to changes in the original DNA copy number (Fig. 1). There may be more or fewer DNA segment copies than in the reference genome [22]. CNVs usually occur in the close proximity of segmental duplications. Segmental duplications are highly identical duplicated sequences (> 90% identity, > 1-kb pairs long) that contribute profoundly to genetic variation in the human genome [23]. They are a dynamic source of genetic variation and have played an important role in the diversification of primates from other apes [23]. During meiosis, segmental duplications can serve as a site for non-allelic homologous recombination which results in the duplication or deletion of a DNA segment [24, 25]. CNVs are usually a consequence of single event or a combination of single events. They can be inherited germline mutations or a consequence of somatic mutations. Indeed, CNVs were identified between twins and within different tissues [24]. Most CNVs in the human genome are benign variants. However, when CNVs affect genes or regulatory regions, they can lead to genetic diseases. The functional consequences of CNVs in gene regions are attributed to gene dosage effects. Duplication or deletion of a gene allele leads to higher/lower gene dosage. Similar to SNPs, the vast majority of CNVs have been identified outside gene regions [26]. The high representation of CNVs in the human genome (12%) suggests their importance for gene regulation [24, 27]. CNVs can be identified by many genomic technologies e.g. fluorescence in situ hybridisation, comparative genomic array hybridisation, single nucleotide polymorphism array, next-generation sequencing (NGS), and long read sequencing technologies [14, 17]. CNVs in causative genes can be directly attributed to phenotypic changes and susceptibility to fracture risks. We focus here on CNVs in nuclear DNA, although variations in the number of mitochondrial DNA have also been associated with osteoporosis [28]. Several causal genes with CNVs have been correlated with fracture risk or BMD [19, 29–33]. The first CNV associated with OP was identified in 2008 [29]. Using the microarray screening approach, the authors discovered CNV 4q13.2, a deletion in the *UGT2B17* (UDP glucuronosyltransferase 2 family, polypeptide B17) gene. UGT2B17 is a glucuronosyltransferase enzyme that plays a role in the metabolism of steroid hormones, including oestrogen and androgen, which are known to be important in bone metabolism. Deng et al. identified a CNV in the *VPS13B* gene in 1000 Caucasians that is highly associated with low BMD [30]. CNVs causing early-onset osteoporosis have been discovered in the gene for collagen type 1, which plays a crucial role in bone metabolism, in patients with osteogenesis imperfecta type I [31]. Chew et al. identified CNVs in the tumour suppressor gene *APC*, which is highly associated with low BMD [33]. APC inhibits Wnt-signalling pathway pivotal for bone regulation. A genome-wide CNV association study in 5178 individuals from a cohort in the Netherlands revealed a 210-kb deletion on chromosome 6p25.1 that was highly associated with OP [32]. This CNV predisposes to a higher risk of fracture only in some European populations.

## CNVs in Causal Genes Directly Affect Development of Osteoporosis

Whole genome sequencing has enabled the identification of novel genetic variations that contribute to complex human diseases, including osteoporosis. In addition, data analysis and the quality of array analyses have improved in recent years, allowing more and more genetic variations to be linked to diseases. Here, we provide an overview of CNVs that have been associated with low BMD or high fracture risk in recent years (Table 1). Cleidocranial dysplasia (CCD) is a rare autosomal dominant skeletal disease caused by mutations in Runt-related transcription factor 2 (*RUNX2*) gene that induce haploinsufficiency in *RUNX2*. RUNX2 is a major transcription factor of osteoblasts development and bone remodelling [35]. Mutations in *RUNX2* have been detected in 60–70% of CCD patients. A variety of genetic variations in *RUNX2* have been discovered, including microdeletions. Recently, a novel exonic microdeletion in the *RUNX2* gene was found in a 5-year-old girl with clavicular hypoplasia, which has been shown to cause cleidocranial dysplasia [36•]. A 11.38-kb microdeletion in 8–9 exon of *RUNX2* gene caused a decrease in the *RUNX2* expression and inhibited osteogenic differentiation in vitro. The patient showed symptoms of CCD including extra teeth, aplasia of clavicles, sloping shoulders, marked calvarial hypomineralisation and osteoporosis. This study confirmed the importance of CNVs in the major regulator of osteoblast development, *RUNX2*, for the development of bone disease.

**Table 1:** Novel genetic loci with CNVs associated with a bone phenotype

Disease/phenotype	New potential loci important for OP	Reference
Bone fragility in young individuals	*ETV1-DGKB*, *AGBL2*, *ATM, RPS6KL1-PGF*, and *SCN4A*	[34•]
KBG syndrome and low BMD, femur fractures	*ANKRD11*, *RPL13*, and* PGN*	[37]
Osteoporotic fractures	*ABO*, *ELMSAN1*, *SIRPA*, *intergenic regions close to the genes*: *SRIP1*, *TMPRSS11E*, *POLR1D*, *LOC100421611*, *lncRNA LINC01260*	[38]
Osteoporosis	*GPR68*	[39•]
Craniosynostosis	*HDAC9 (containing TWIST1 enhancer sequence)*	[40]

Finnish study searched for rare CNVs in 70 young individuals with skeletal fragility by a custom-made high-resolution comparative-genomic hybridisation (CGH) microarray with increased probe density in more than thousand genes important for bone metabolism [34•]. Among 14 rare CNVs identified, five CNVs affected coding regions of genes that were not previously associated with bone fragility (*ETV1-DGKB*, *AGBL2*, *ATM*, *RPS6KL1-PGF,* and *SCN4A*) [34•]. They also found novel CNVs in the genes previously implicated in bone metabolism, *COL1A2* and *PLS2*. A 4-kb deletion of exons 1–4 in *COL1A2* and a 12.5-kb duplication in exon 3 of the *PLS3* gene were detected in patients with severe osteoporosis, confirming the monogenic nature of both genes in early-onset OP. The mutation in *COL1A2* resulted in a deletion of the pre-prepeptide, reducing protein secretion into the endoplasmic reticulum. Since other genetic variants (SNPs or other mutations) could also contribute to the phenotype, the authors ruled out this possibility by sequencing the whole genome to confirm the significance of the newly identified CNV [34•]. How the newly identified loci correlate with the development of disorders remains to be investigated.

In a case study of Romanian 7-year-old girl with severe developmental delay, intellectual disability, facial dysmorphism, femur fracture and very low bone mineral density, the authors have identified a 113-kb duplication that encompassed several genes [37]. This region included ankyrin repeat domain-containing protein 11 (*ANKRD11* (exon1)), *RPL13* and *PGN* [37].

Other genetic variants that could contribute to severe bone phenotype were excluded by exome sequencing. [37]. *ANKRD11* regulates transcription via inhibition of histone acetylase and was previously demonstrated to play an important role in skeletal and central nervous system development in KBG syndrome patients [41]. Experiments on mice have shown that mutations in C-terminal domain of ANKRD11 eliminate their inhibitory effect on the gene expression and cause reduced BMD [42]. Whether promoter duplication in *ANKRD11* gene leads to modulated gene expression and accounts for decrease in BMD awaits further functional validation. RPLP13 is a component of 60S ribosomal subunit, and its high expression has previously been associated with an autosomal dominant disorder Isidor-Toutain spondyloepimetaphyseal dysplasia [43], suggesting that duplication in *RPLP13* could directly affect low BMD.

In a study of familial and idiopathic OP, Rocha-Braz and colleagues aimed to find novel genetic variants associated with low BMD and development of OP [39•]. They targeted sequenced a selection of 128 bone-related genes or genes of unknown significance yet previously associated with low BMD. Their cohort included 37 patients with idiopathic or familial OP that showed development of OP at a younger age and had prevalence for fractures. They discovered 28 genetic variations, one quarter of them resided in known bone genes (e.g. *COL1A2*, *WNT1*, *IDUA*, *PLS3* and *NOTCH2*). In addition, a deletion of a 41.5-kb region that included exon 2 of *GPR68* gene was discovered in a male that developed OP at the age of 42 years [39•]. *GPR68* is a proton-sensing G-protein-coupled receptor that responds to extracellular pH and regulates various cellular functions. Mice deficient in GPR68 showed reduced osteoclast differentiation, abnormalities in osteoclastogenesis and decreased tumorigenesis which indicated that GPR68 plays a role in bone remodelling [39•]. Indeed, a homozygote detrimental mutation in the human GPR68 gene was associated with altered enamel mineralisation in human amelogenesis imperfecta [44].

An example of a loss-of-function mutation was discovered in patients with trichorhinophalangeal syndrome (TRPS). TRPS is a rare autosomal dominant disorder characterised by craniofacial and skeletal abnormalities. TRPS I is caused by a variety of mutations in the *TRPS1* gene, including a gene deletion [45]. A recent study described a novel genetic variant in the *TRPS1* gene that causes syndromic brachydactyly with defects in skeletal formation and growth plate development [46]. In addition, GWAS studies have found an association of SNPs in the *TRPS1* gene with femoral neck bone mineral density [47] and estimated heel bone mineral density, indicating the importance of the gene in bone remodelling [48].

## CNVs in Regulatory Regions Can Modulate Gene Expression of Osteoporotic Genes

CNVs residing in regulatory regions (enhancers and promoters) and long non-coding RNA can modulate gene expression of OP-related genes. In a comprehensive study by Hirsch et al., structural variations in *HDAC9* gene that influenced the transcription of the neighbouring *TWIST1* gene were discovered in craniosynostosis patients [40]. Deletions in *HDAC9* gene, but not in the *TWIST1* protein-coding sequence, caused development of craniosynostosis. Regulatory elements that reside in the *HDAC9* gene contributed to the transcriptional regulation of the neighbouring craniofacial gene *TWIST1* [40]. Deletion of *TWIST1* enhancers within the *HDAC9* gene induced a small size skull in their mouse model, confirming the functional role of the regulatory region of the *TWIST1* gene. *TWIST1* is a transcription factor important for mesodermal development [40].

A genome-wide CNV association study of 1537 Koreans revealed 8 CNV regions highly associated with osteoporotic fractures [38]. Using CGH arrays the authors identified CNV loci in (a) the intergenic regions close to the genes: *SRIP1*, *TMPRSS11E,* *POLR1D* and *LOC100421611*; (b) a non-coding RNA LINC01260; and (c) the intragenic region of the gens: *ABO*, *ELMSAN1* and *SIRPA* [38]. A deletion on chromosome 20q13.12in close proximity to the LINC01260 was confirmed by a Q-PCR method [38]. All of the identified genetic variants still need to be functionally validated.

Since CNVs in non-coding or regulatory regions indirectly influence a phenotype, they are much more difficult to identify and evaluate. Therefore, the identified CNV loci likely represent only a small fraction of the CNVs that have shaped the landscape of OP-related genes. Comprehensive analyses involving multiple computational and technological approaches will yield many more disease-related CNVs in the future.

The search for new therapeutic targets is mostly focused on identifying new gene targets. However, regulatory regions and non-coding RNAs (ncRNAs) also represent opportunities for targeted therapies. In particular, ncRNAs have great potential for drug targets due to their ability to regulate gene expression and disease progression [49]. Once a specific ncRNA target is identified, it can be effectively and selectively targeted with a complementary oligonucleotide. The discovery of CNVs in regulatory regions could provide new therapeutic targets for the treatment of low bone density. Since ncRNA can regulate the expression of multiple genes, the mechanisms of a particular regulatory element should be thoroughly deciphered before it is used for targeted therapy.

## Conclusion

Recent studies of genetic variants have identified genes (e.g. *TWIST1*, *GPR68* and *ANKRD11)* that were not thought to play a role in bone remodelling. However, most of the recently discovered CNVs associated with low BMD or osteoporotic fractures belong to intragenic regions. The majority of CNVs in genomes reside in the intergenic regions and are difficult to associate with pathology. Therefore, additional studies are needed to identify CNVs in the intergenic regions. For example, analysis of CNVs in the telomeric region using data from the gnomAD-SV database [16] may reveal new bone-related CNVs. The completion of the human genome sequence T2T (telomere to telomere)-CHM13 [23] also offers new opportunities for the identification of segmental duplications and CNVs related to bone-associated genes. In addition, the role of somatic mutations (including CNVs) in osteoporosis remains an open field for future bone research. Although the rapid development and accessibility of NGS methods is helping to identify new CNVs, it also has its pitfalls. One of the major challenges in researching novel CNVs is the high range of variability of WGS data, which leads to noise in the sequencing data. The future of research therefore lies in an integrative approach in which SNPs and CNVs (GWAS and WGS data) are analysed together and associated with phenotypes. For example, a novel CNEST bioinformatics tool promises to find new functional genetic variants by combining large-scale GWAS and WGS data [50]. A recently described tool, ‘CNV-espresso’, has been developed to exploit CNVs from exome sequences, which will also help identify new genetic variants associated with disease [51•] .

Although promising software tools and the development/accessibility of sequencing methods will yield more CNVs associated with low BMD or increased risk of fragility fractures, each genetic variant needs to be confirmed by a different molecular biology method and validated for functional significance.

## Data Availability

This is a review paper and no raw data were included in the manuscript.
